# Hypersensitivity in the lungs is responsible for acute respiratory failure in COVID‐19 patients: Case series of patients who received high‐dose/short‐term methylprednisolone

**DOI:** 10.1002/clt2.12056

**Published:** 2021-08-27

**Authors:** Meizhu Chen, Changli Tu, Cuiyan Tan, Xiaobin Zheng, Fengfei Sun, Yingjian Liang, Honglei Shi, Jian Wu, Yiying Huang, Zhenguo Wang, Kongqiu Wang, Minmin Lin, Weiming Wu, Hong Zhou, Jing Liu, Jin Huang

**Affiliations:** ^1^ Department of Pulmonary and Critical Care Medicine (PCCM) The Fifth Affiliated Hospital of Sun Yat‐sen University Zhuhai Guangdong Province China

**Keywords:** anti‐SARS‐CoV‐2 specific IgE, COVID‐19, hypersensitivity, hypoxic respiratory failure, methylprednisolone

## Abstract

**Background:**

COVID‐19 is a highly contagious respiratory disease caused by the SARS‐CoV‐2 virus. Patients with severe disease have a high fatality rate and face a huge medical burden due to the need for invasive mechanical ventilation. Hypoxic respiratory failure is the major cause of death in these patients. There are currently no specific anti‐SARS‐CoV‐2 drugs, and the effect of corticosteroids is still controversial.

**Methods:**

The clinical data of 102 COVID‐19 patients, including 27 patients with severe disease, were analyzed. The serum levels of total IgE and anti‐SARS‐CoV‐2 specific IgE were compared in healthy controls and COVID‐19 patients, changes in the level of anti‐SARS‐CoV‐2 specific IgE and clinical response to methylprednisolone (MP) treatment were analyzed, and the effect of high‐dose/short‐term MP therapy for patients with critical illness and respiratory failure was determined.

**Results:**

COVID‐19 patients had elevated serum levels of anti‐SARS‐CoV‐2 specific IgE, and patients with severe disease, especially critical illness, had even higher levels. Application of short‐term/high‐dose MP significantly reduced the level of these IgE antibodies and also blocked the progression of hypoxic respiratory failure. Hypoxic respiratory failure in patients with COVID‐19 is related to pulmonary hypersensitivity.

**Conclusions:**

Hypersensitivity in the lungs is responsible for acute respiratory failure in COVID‐19 patients. Application of high‐dose/short‐term MP appears to be an effective life‐saving method for COVID‐19 patients who have hypoxic respiratory failure.

## 1 BACKGROUND

Studies of COVID‐19 patients should seek to more fully understand the regularity of different signs and symptoms and to identify the major causes of death. Most studies reported that the major cause of death in critically ill patients with COVID‐19 pneumonia was hypoxic respiratory failure. These patients experience death from severe ventilation dysfunction, rather than the structural lung damage caused by the inflammation itself.^1^, ^2^, ^3^, ^4^ There are currently no specific anti‐SARS‐CoV‐2 drugs, and the benefit of corticosteroids therapy is controversial. Two meta‐analyses of prospective clinical trials of patients who were critically ill with COVID‐19 reported that the use of systemic corticosteroids (compared with usual care or placebo) was associated with a reduced 28‐day all‐cause mortality and an increased number of ventilator‐free days.^5^, ^6^ But another clinical study of patients who were critically ill with COVID‐19 and had acute respiratory failure reported that low‐dose hydrocortisone (compared with placebo) did not significantly reduce treatment failure, defined as death or persistent respiratory support at day 21.^7^

Our research team successfully treated 102 patients who were hospitalized with COVID‐19 pneumonia from January 2020 to April 2020. These patients included 27 patients with severe disease, 16 of whom had critical illness. There were no deaths or complications after treatment, and all patients were successfully recovered and discharged.

Based on our treatment experience and further in‐depth research, we believe that some healthy adults are pre‐sensitized to SARS‐CoV‐2‐related antigens and that this occurred before the onset of COVID‐19.^8^, ^9^ We propose that the mechanism of critical COVID‐19 pneumonia is hypoxic respiratory failure caused by hypersensitivity of the lungs and that appropriate use of methylprednisolone (MP), such as high‐dose/short‐term therapy, can inhibit the progression to respiratory failure and provide satisfactory results.

## 2 METHODS

### 2.1 Data sources

This study included 102 patients with confirmed COVID‐19 who were older than 14 years and were admitted to the Fifth Affiliated Hospital of Sun Yat‐sen University from January 17, 2020 to April 26, 2020. There were 75 patients with non‐severe disease and 27 with severe disease (16 of whom had critical disease). The healthy control group consisted of 50 people who came to our hospital for physical examinations during the same time period. This study was approved by the Research Ethics Committee of The Fifth Affiliated Hospital of Sun Yat‐sen University (approval series number K167‐1). Clinical data were from the electronic medical records and included basic demographic data, symptoms, vital signs, clinical classifications, complications, and clearance time of SARS‐CoV‐2 RNA from nasopharyngeal swabs (defined as the two consecutive negative real‐time polymerase chain reaction (PCR) results that were 1‐day apart).

### 2.2 SARS‐CoV‐2 nucleic acid test

After admission, respiratory specimens from the nasopharynx and/or throat were routinely collected at 1 to 3‐day intervals. Samples were assessed using an reverse transcription‐polymerase chain reaction (RT‐PCR) assay to confirm SARS‐CoV‐2 infection and were also screened for common respiratory pathogens, such as influenza A and B.^10^ Initially, the RT‐PCR tests were conducted by Zhuhai Center for Disease Control and Prevention; after February 24, 2020, tests were conducted in our clinical laboratory using a SARS‐CoV2 Kit (Shanghai ZJ Bio‐Tech Co., Ltd) with the Applied Biosystems 7500 Real‐Time PCR instrument. After collection, the nasal and throat swabs were put together into a collection tube containing 2.0 ml of viral transport medium, and SARS‐CoV‐2 RNA was extracted within 2 h using a nucleic acid extraction kit (DAAN Gene Co., Ltd). The concentration of harvested RNA was determined using a Nanodrop 2000 spectrophotometer, and the extracted product was used for a real‐time reverse‐transcription PCR assay for SARS‐CoV‐2. The three target genes, RNA‐dependent RNA polymerase (RdRP), envelope (E), and nucleocapsid (N), were simultaneously amplified according to the manufacturer's protocol using the following primers and probes: RdRP: forward primer GTGARATGGTCATGTGTGGCGG; reverse primer CARATGTTAAASACACTATTAGCATA; probe: CAGGTGGAACCTCATCAGGAGATG C‐BHQ1. E: forward primer ACAGGTACGTTAATAGTTAATAGCGT; reverse primer ATATTGCAGCAGTACGCACACA; probe: ACACTAGCCATCCTTACTGCGCTTCG‐BHQ1. N: forward primer CACATTGGCACCCGCAATC; reverse primer GAGGAACGAGAAGAGGCTTG; probe: ACTTCCTCAAGGAACAACATTGCCA‐BHQ1.

The real‐time RT‐PCR assay was performed according to the manufacturer's protocol (Shanghai ZJ Bio‐Tech Co., Ltd). The reaction mixture contained 2 μl of reaction buffer, 1 μl of enzyme solution, 3 μl of probe primers, 4 μl of diethylpyrocarbonate‐treated water, and 5 μl of RNA template. The following amplification procedure was used: incubation at 45°C for 10 min and then 95°C for 3 min; 45 cycles of denaturation (95°C for 15 s); and then extension/annealing (58°C for 30 s). Fluorescence was then measured. A sample with a cycle threshold (Ct) value less than 40 was considered as positive, and those with a Ct value of 40 or more as negative.

### 2.3 Total serum IgE test

The sandwich method was used to measure total serum IgE by electrochemiluminescence (Human IgE ELISA kit, Roche Diagnostics GmbH). First, 10 μL of a peripheral blood sample, a biotinylated anti‐IgE monoclonal antibody, and a ruthenium (Ru)‐labeled anti‐IgE monoclonal antibody were mixed to form a sandwich complex. Then, streptavidin‐coated microparticles were added and the complex formed through the reaction between biotin and streptavidin. This reaction mixture was sucked into the measuring cell, the particles are adsorbed onto the electrode by the magnet, and the unbound substance was washed away using a cleaning solution. Then, a voltage was applied to the electrode to produce chemiluminescence, which was measured by a photomultiplier. The machine automatically calculated the results from a standard curve. The instrument uses a two‐point calibration method, obtained by scanning the reagent barcode or electronic barcode into the original standard curve.

### 2.4 Anti‐SARS‐CoV‐2 specific IgE test

An enzyme‐linked immunoabsorbent assay (ELISA) was used to measure anti‐SARS‐CoV‐2 specific IgE. First, a 96‐well microtiter ELISA plate was coated with 2 μg ml^−1^ of recombinant SARS‐CoV‐2 S antigen and SARS‐CoV‐2 N antigen (Sino Biological) overnight at 4°C with blocking by 20% nonfat dried milk. Then, samples were added and incubated at 37°C for 2 h, and the plate was then washed three times with phosphate buffered saline containing 0.04% Tween‐20. For measurement of IgE, 50 μl of serum (not diluted in phosphate buffer saline (PBS) was incubated at 37°C for 2 h. Then, 100 μl of horse radish peroxidase (HRP) labeled goat anti‐human IgE HRP (diluted 1:5000; Abcam, ab3901) was added, and the sample was incubated at room temperature for 2 h. The plates were washed three times with PBS, and the signal was developed by adding 100 μl of the 3,3',5,5'‐ tetramethylbenzidine substrate (Solarbio) for 15 min at room temperature. The reaction was stopped by adding 50 μl of 2‐M sulfuric acid. Plates were read on a Spectramax Plate Reader at 450 nm using SoftMax Pro, and the background optical density was subtracted. The cutoff (normal) value was the average level in the healthy controls.

### 2.5 Statistical analysis

Categorical variables were expressed as numbers and percentages, and continuous variables were expressed as means and standard deviations or as medians or interquartile ranges. Mean values were compared using an independent samples *t‐*test and a one‐way analysis of variance when the data were normally distributed and homoscedastic. The Mann–Whitney *U* test and the Kruskal–Wallis *H* test were used to compare data that had non‐normal distributions. The proportions for categorical variables were compared using the chi‐squared test, but Fisher's exact test was used when the number of data was limited. All statistical analyses were performed using SPSS version 26.0 (IBM). For unadjusted comparisons, a two‐sided *p*‐value below 0.05 was considered significant.

## 3 RESULTS

### 3.1 Demographic and clinical characteristics at admission

We included 102 patients with confirmed COVID‐19 who were treated by our medical team, 27 of whom (26.5%) had severe disease (Table 1). Ninety‐seven patients were local residents of China and five patients were from England. The 50 healthy controls were all local residents of China. The major clinical symptoms were fever, shortness of breath, cough, diarrhea, loss of appetite, hemoptysis, fatigue, nasal congestion, runny nose, chest tightness, and sputum production. Comparison of patients with severe versus non‐severe disease indicated that hemoptysis (25.9% vs. 0, *p* < 0.001), shortness of breath (100% vs. 9.3%, *p* < 0.001), and fever (81.5% vs. 49.3%, *p* = 0.006) were more common in the severe disease group. However, the two groups had no significant differences in underlying diseases (all *p* > 0.05). The non‐severe group had no patients with Sequential Organ Failure Assessment (SOFA) scores above 9 and no patients who experienced shock. However, the severe group had five patients with SOFA scores greater than 9 (*p* = 0.001), two patients with SOFA scores greater than 11, and four patients who experienced shock (*p* = 0.005).

**TABLE 1:** Demographic and clinical characteristics of 102 patients with COVID‐19 with severe or non‐severe disease and 50 healthy controls

	All patients	Non‐severe group	Severe group	Healthy controls		
Clinical characteristic	(*n* = 102)	(*n* = 75)	(*n* = 27)	(*n* = 50)	*p*‐Value*	*p*‐Value**
Age, median (range), years	47.6 (15–80)	41.0 (15–75)	61.0 (32–80)	49.0 (25–76)	0.056	
Sex						
Female, *N* (%)	57/102 (55.9)	46/75 (61.3)	11/27 (40.7)	24/50 (48.0)	0.121	
Male, *N* (%)	45/102 (44.1)	29/75 (38.7)	16/27 (59.3)	26/50 (52.0)		
Smoking history, *N* (%)	8/102 (7.8)	5/75 (6.7)	3/27 (11.1)	4/50 (8.0)	0.525	
Coexisting disorders, *N* (%)						
Hypertension	16/102 (15.7)	10/75 (13.3)	6/27 (22.2)	8/50 (16.0)	0.571	
Type 2 diabetes mellitus	6/102 (5.9)	2/75 (2.7)	4/27 (14.8)	5/50 (10.0)	0.069	
Malignancy	6/102 (5.9)	4/75 (5.3)	2/27 (7.4)	2/50 (4.0)	0.82	
Respiratory symptoms, *N* (%)						
Fever	59/102 (57.8)	37/75 (49.3)	22/27 (81.5)			0.006
Hemoptysis	7/102 (6.9)	0/75 (0)	7/27 (25.9)			<0.001
Shortness of breath	34/102 (33.3)	7/75 (9.3)	27/27 (100.0)			<0.001
Asymptomatic on admission	12/102 (11.8)	12/75 (16.0)	0/27 (0)			0.062
Laboratory findings						
WBC count, mean (±SD), ×10^9^ L^−1^	4.1 ± 1.5	4.3 ± 0.2	3.7 ± 0.3			0.082
LAC, median (range), mmol L^−1^	2.3 (2.2–2.6)	2.3 (2.2–2.7)	2.4 (2.3–2.7)			0.237
Shock, *N* (%)	4/102 (3.9)	0/75 (0)	4/27 (14.8)			0.005
SOFA score > 9, *N* (%)	5/102 (4.9)	0/75 (0)	5/27 (18.5)			0.001

Abbreviations: LAC, lactic acid; SD, standard deviation; SOFA, Sequential Organ Failure Assessment; WBC, white blood cell.

*Significant difference between COVID‐19 patients and healthy controls.

**Significant difference between the non‐severe and severe groups.

### 3.2 Blood test results

**FIGURE 1:**
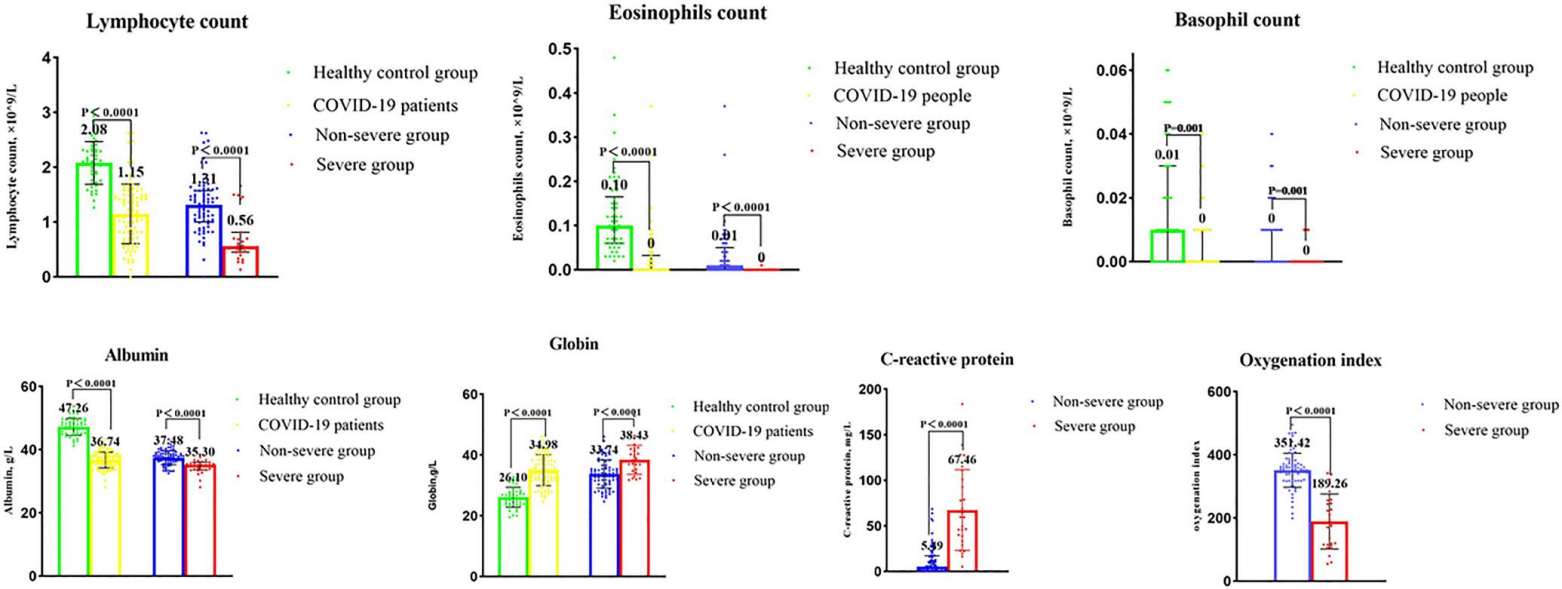
Counts of lymphocytes, eosinophils, and basophils; levels of serum albumin, immunoglobulin, and c‐reactive protein; and oxygenation indexes of patients in the different groups

We analyzed the blood data of the controls and patients (Figure 1). Comparisons of the granulocyte composition of all 102 COVID‐19 patients with healthy controls indicated that the patients had lower levels of lymphocytes, eosinophils, and basophils (all *p* < 0.05). Furthermore, the severe disease group had lower levels of all three cell types than the non‐severe disease group (all *p* < 0.05). Relative to the healthy controls, COVID‐19 patients also had lower levels of albumin and higher levels of immunoglobulin (both *p* < 0.05); relative to the non‐severe disease group, the severe disease group had a lower level of albumin and a higher level of immunoglobulin (both *p* < 0.05). Relative to the non‐severe disease group, the severe disease group had a higher level of c‐reactive protein and a lower oxygenation index (both *p* < 0.05).

### 3.3 Anti‐SARS‐CoV‐2 specific IgE levels

**FIGURE 2:**
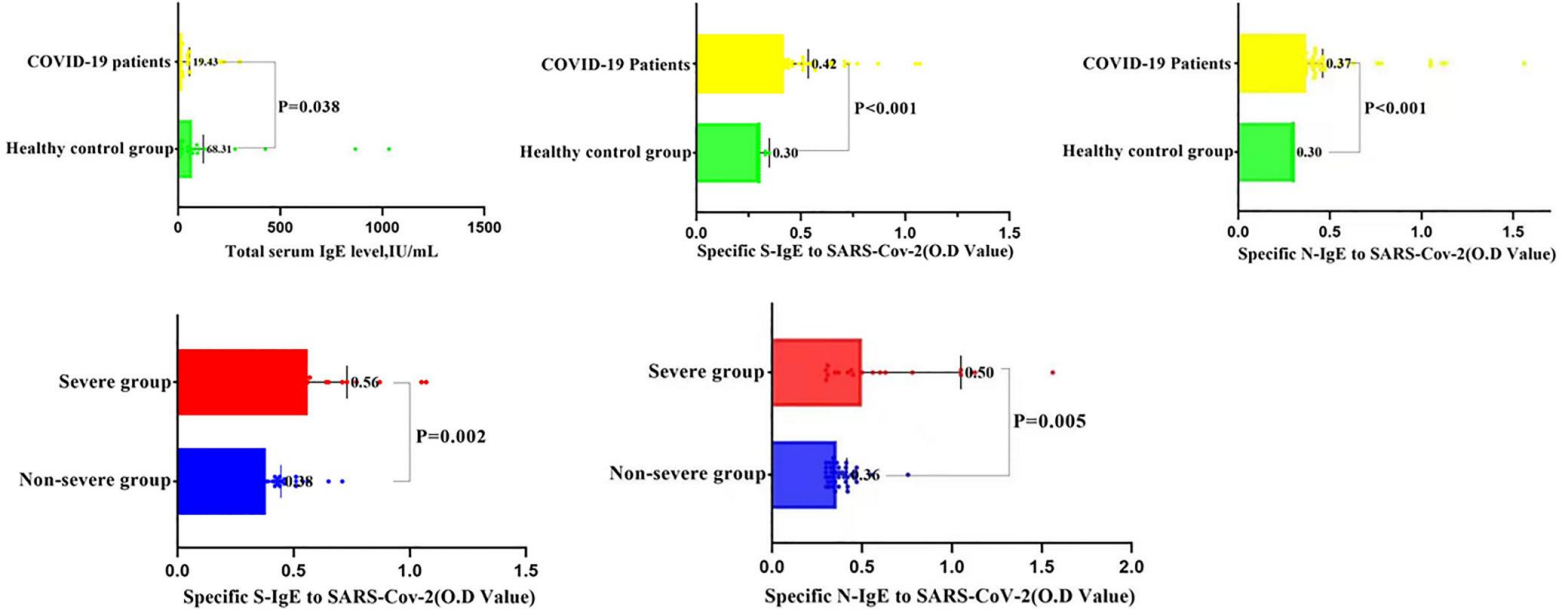
Levels of the total serum IgE, two anti‐SARS‐CoV‐2 specific IgE (anti‐S IgE and anti‐N IgE) in healthy controls and COVID‐19 patients. The total IgE level of the healthy control group was significantly higher than that of COVID‐19 patients. The COVID‐19 patients had significantly elevated levels of two anti‐SARS‐CoV‐2 specific IgE (anti‐S IgE and anti‐N IgE). Further analysis found that the levels of these antibodies were higher in severe disease patients than in non‐severe disease patients

Our measurements of total serum IgE indicated that the healthy controls had higher levels than the COVID‐19 patients (63.81 vs. 19.43 IU ml^−1^, *p* = 0.038; Figure 2). We do not yet know the reason for the lower total serum IgE level in COVID‐19 patients, but this is an intriguing result. Measurements performed at admission also indicated that the COVID‐19 patients had significantly elevated levels of two anti‐SARS‐CoV‐2 specific IgE (anti‐S IgE: 0.42 vs. 0.3, *p* < 0.001; anti‐N IgE: 0.37 vs. 0.3, *p* < 0.001; Figure 2). Further analysis found that the levels of these antibodies were higher in severe disease group than in non‐severe disease group (anti‐S IgE: 0.56 vs. 0.38, *p* = 0.002; anti‐N IgE: 0.5 vs. 0.36, *p* = 0.005; Figure 2). The higher levels of these antibodies in severe disease patients thus correlate with the presence of pulmonary hypersensitivity.

### 3.4 Efficacy of MP in treatment of COVID‐19 hypoxic respiratory failure

**FIGURE 3:**
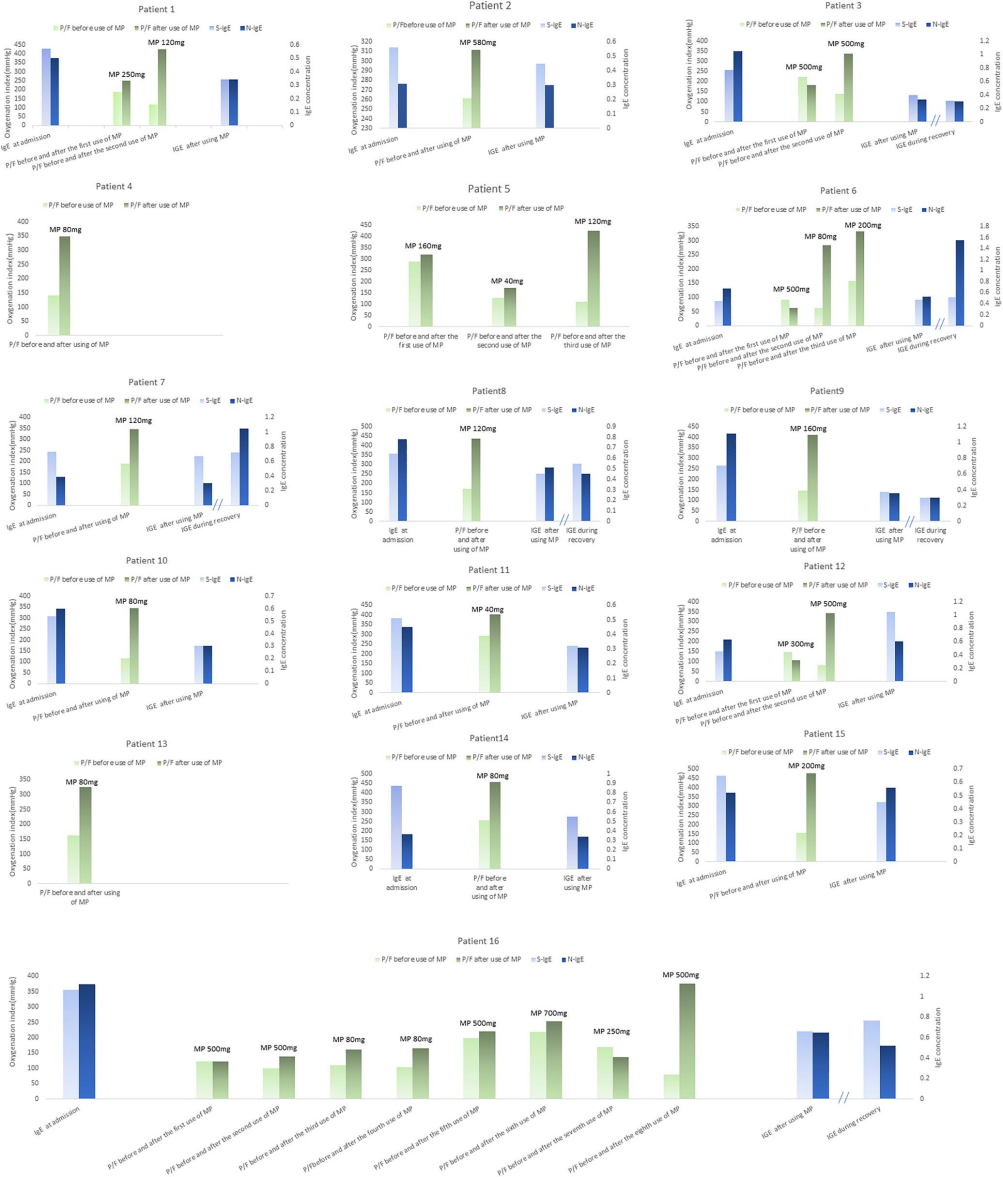
Analysis of the course of disease in 16 patients with critical COVID‐19 pneumonia. Blue bars indicate anti‐S IgE and anti‐N IgE and green bars indicate oxygenation index immediately before and after the administration of MP. Note that six patients received multiple pulse therapies of methylprednisolone (MP). In all cases, MP was readministered if a patient's oxygenation index remained below 300 mmHg and there was no significant resolution of dyspnea symptoms

Notably, each of the 16 critically ill patients experienced clinically significant improvements in the lung oxygenation index after each administration of intravenous drip MP (Figure 3). Notably, patient #16 received eight doses of MP (ranging from 80 to 700 mg) before restoration of lung function and discharge on day 23. Patients with critical COVID‐19 pneumonia experience hypoxic respiratory failure, suggesting that clinical interventions, which reduce the onset and persistence of hypoxia, are key to treating these patients. Our experience is that a patient with an oxygenation index of 150 mmHg or less should receive intravenous drip MP at a dose of 4–8 mg kg^−1^ day^−1^ to block the hypersensitivity reaction in the lungs. Our results (Figure 3) indicated that application of MP significantly increased the PaO_2_/FiO_2_ ratio and led to resolution of respiratory failure. In these patients, we started at a median dose of 1–2 mg kg^−1^ day^−1^, and if the effect was not satisfactory, we increased it to 3–4 mg kg^−1^ day^−1^. Patient 16 received a total of 3.11 g of MP but experienced no corticosteroid‐related adverse effects based on a telephone follow‐up after 1 year. We found that the level of anti‐SARS‐CoV‐2 specific IgE before MP was significantly higher than after MP, although there were some increases of IgE during the recovery period.

### 3.5 Effect of MP on the time to SARS‐CoV‐2 nucleic acid negativity

**FIGURE 4:**
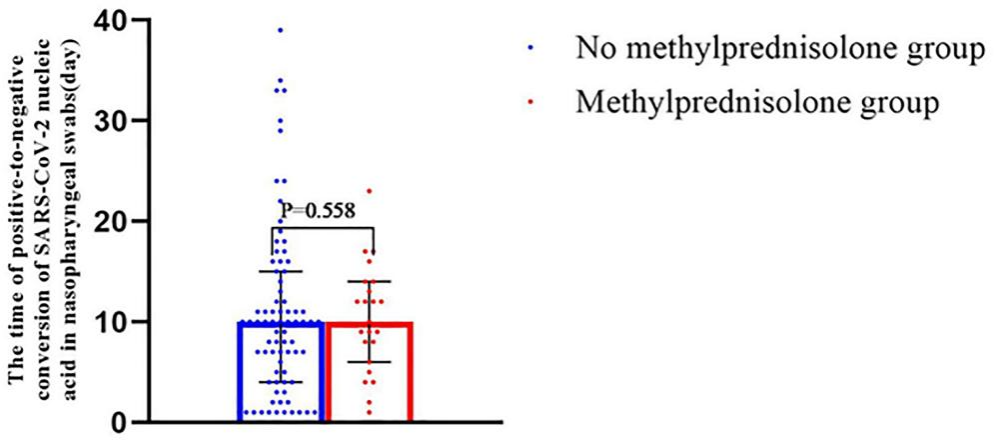
Relationship between methylprednisolone (MP) use and time from positive‐to‐negative conversion of SARS‐CoV‐2 nucleic acid in nasopharyngeal swabs in no MP group (*n* = 86) versus MP group (*n* = 16)

Among all the 102 patients, our comparison of patients who did and did not receive MP indicated no significant difference in the mean time to SARS‐CoV‐2 nucleic acid negativity (10.3 days vs. 10.7 days; Figure 4).

**FIGURE 5:**
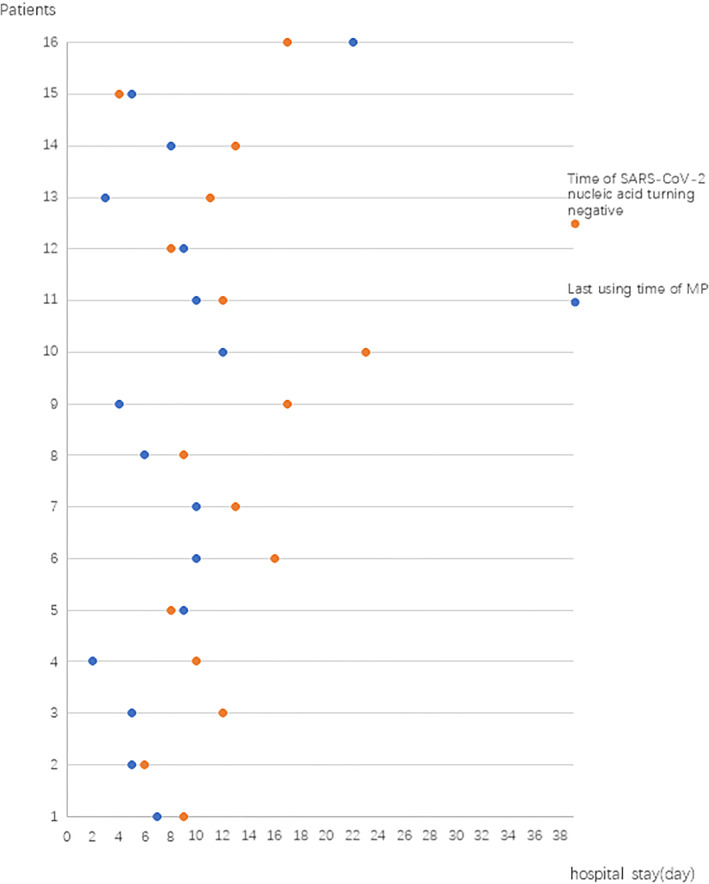
Relationship between the day of the last use of methylprednisolone with the day of SARS‐COV‐2 nucleic acid positive‐to‐negative conversion in nasopharyngeal swabs for the 16 patients with critical disease

We also analyzed the relationship of the date to SARS‐CoV‐2 nucleic acid negativity and the date of last use of MP in patients with critical illness (Figure 5). These results indicated that the SARS‐CoV‐2 nucleic acid negative conversion time was very close to the time when MP use ended. In particular, our data showed that the SARS‐CoV‐2 nucleic acid negativity occurred an average of 3.8 days after stopping use of MP in critical COVID‐19 patients. We believe that SARS‐CoV‐2 nucleic acid negativity may be an indicator of elimination of the initiating factor of hypersensitivity in the lungs and that no further MP treatment is needed.

**FIGURE 6:**
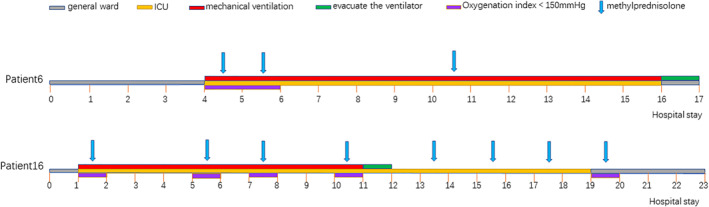
Treatment of two patients with critical disease who were admitted to the intensive care units (ICU), received mechanical ventilation and methylprednisolone, and were then weaned from oxygen therapy, discharged from the ICU, and then discharged from the hospital

Among the 16 critically ill COVID‐19 pneumonia patients who received MP, patient #6 and patient #16 received tracheal intubation with ventilator assistance on day 1 (patient #16) and on day 4 (patient #6) after admission due to unsatisfactory respiratory control (Figure 6). After 10 days (patient #16) and 12 days (patient #6) of mechanical ventilation and several MP treatments, both patients fully recovered.

## 4 DISCUSSION

One of the main functions of IgE in allergic responses is in type I hypersensitivity, and this immunoglobulin is a necessary starting factor during hypersensitivity reactions.^11^ Critical COVID‐19 pneumonia is characterized by the rapid progression of dyspnea.^12^ Radiological and pathological examinations of patients with COVID‐19 pneumonia indicate the presence of severe inflammatory reactions in the lungs that resemble hypersensitivity pneumonitis. This distinguishes COVID‐19 from other types of pneumonia.^13^, ^14^, ^15^

There were 102 patients with COVID‐19 pneumonia who were admitted to our institution from January 17, 2020 to April 26, 2020. Our research team successfully treated all 102 patients, including the 16 patients who were critically ill. In all cases, our interventions for these critically ill patients (high‐dose/short‐term MP) were designed assuming the presence of hypersensitivity pneumonitis, and we achieved excellent results, with 100% recovery and no fatalities.

Our results indicated the presence of elevated levels of anti‐SARS‐CoV‐2‐specific IgE in COVID‐19 patients and that the levels of these antibodies were higher in severe disease patients than in non‐severe disease patients. In addition, after treatment of critically ill patients with MP, the levels of anti‐SARS‐CoV‐2 IgE significantly decreased in parallel with the improvements of the lung oxygenation index. Our healthy controls had almost no anti‐SARS‐CoV‐2 specific IgE, and the levels were significantly lower than the COVID‐19 pneumonia group. Thus, based on our treatment experience, we propose that severe COVID‐19 pneumonia begins after SARS‐CoV‐2 infection initiates a specific IgE‐mediated, type I immediate hypersensitivity reaction in the lungs. Therefore, we recommend that when this lung hypersensitivity leads to hypoxic respiratory failure, MP (medium‐ or high‐dose) should be administered to inhibit the progression of hypersensitivity and prevent respiratory failure. Even patients who require mechanical ventilation should receive high doses of MP at an appropriate time to promote weaning from the ventilator as soon as possible.^16^

Based on the concepts developed for treatment for hypersensitivity, we do not recommend the use of corticosteroids for treatment of COVID‐19 pneumonia when given in small doses and for a long period of time (e.g., 10 days), because this treatment may suppress the body's natural immune state and impede SARS‐CoV‐2 removal. Instead, based on our clinical experiences and previous research, short‐term medium or high doses of MP does not lead to complications and does not affect the body's clearance of SARS‐CoV‐2.^17^, ^18^

## 5 CONCLUSIONS

The main cause of death from critical COVID‐19 pneumonia may be the hypersensitivity reaction that occurs in the lungs. Our successful treatment of 16 patients who had critical COVID‐19 pneumonia by use of high‐dose/short‐term MP is consistent with the presence of lung hypersensitivity. Thus, for patients with critical COVID‐19 pneumonia, we recommend use of short‐term medium or high doses of MP as the main treatment. We recommend against continuous low‐dose corticosteroids therapy for these patients to avoid downregulation of immune functions.

## CONFLICT OF INTEREST

The authors of this study declare no relationships with any companies whose products or services might be related to the subject matter of the article.

## AUTHOR CONTRIBUTIONS

*Conceptualization and design*: Jin Huang, Jing Liu, Meizhu Chen, Changli Tu, and Cuiyan Tan. *Data curation and investigation*: Meizhu Chen, Changli Tu, Cuiyan Tan, Xiaobin Zheng, Fengfei Sun, Yingjian Liang, Honglei Shi, Jian Wu, Yiying Huang, Zhenguo Wang, Kongqiu Wang; Minmin Lin, Weiming Wu, and Hong Zhou. *Formal analysis*: Meizhu Chen, Changli Tu, Jin Huang, and Jing Liu. *Funding* *acquisition*: Jing Liu and Jin Huang. *Methodology*: Jin Huang, Jing Liu, Meizhu Chen, Changli Tu, and Cuiyan Tan. *Project administration and supervision*: Jin Huang and Jing Liu. *Writing—original draft*: Meizhu Chen, Changli Tu, and Cuiyan Tan. *Writing—review and editing*: Jin Huang and Jing Liu.
